# Analysis of Risk Factors and Development of a Diagnostic Model for Ischemia in Patients With Nonobstructive Coronary Artery Disease Based on Positron Emission Tomography/Computed Tomography

**DOI:** 10.31083/RCM48741

**Published:** 2026-08-13

**Authors:** Rui Jing, Wei Zhu, Yanmin Zhou, Jinglin Sun, Huan Luo, Ruijuan Fan, Ning Yang, Maoti Wei, Yuming Li

**Affiliations:** ^1^Department of Cardiology, TEDA International Cardiovascular Hospital, Tianjin University, 300457 Tianjin, China; ^2^Clinical College of Cardiovascular Diseases, Tianjin Medical University, 300457 Tianjin, China; ^3^Department of Radiology and Nuclear Medicine, Tianjin Second People’s Hospital, 300192 Tianjin, China; ^4^Department of Radiology and Nuclear Medicine, Clinical School of the Second People’s Hospital, Tianjin Medical University, 300192 Tianjin, China; ^5^Department of Cardiology, Tianjin Kanghui Hospital, 300380 Tianjin, China

**Keywords:** nonobstructive coronary artery disease, coronary microcirculation, myocardial blood flow reserve, positron emission tomography, random forest diagnostic model

## Abstract

**Background:**

This study aimed to identify search for clinical indicators of ischemia with no obstructive coronary arteries (INOCA) and to develop a preliminary diagnostic and predictive model to identify high-risk patients in clinical practice.

**Method:**

(1) We retrospectively reviewed patients admitted to the hospital for chest pain or related symptoms who were clinically suspected of having angina pectoris. Patients who received complete nuclear coronary flow reserve (CFR) measurements obtained by positron emission tomography/computed tomography (PET/CT) quantitative coronary artery assessment, had undergone coronary angiography, and had no obstructive coronary lesions were selected as study subjects. Based on the CFR results, patients with a minimum CFR <2.0 were classified as the INOCA group (diagnosed group), and those with a minimum CFR ≥2.5 were classified as the non-INOCA group (control group). Clinical indicators were compared between the two groups. A multiple logistic regression analysis was performed to identify high-risk factors and to establish an INOCA diagnostic model. Additionally, a random forest INOCA diagnostic model based on decision trees was constructed using machine learning methods. (2) An independent cohort of patients meeting the same inclusion criteria as in Method (1) was used for external validation and evaluation of the previously established multiple logistic regression and random forest models.

**Results:**

(1) During the model development phase, the results of the multiple logistic regression analysis indicated that smoking history, standard deviation of normal R-R interval (SDNN), low-density lipoprotein-cholesterol (LDL-C), hematocrit (HCT), and monocyte absolute value (MONO#) may be associated with the risk of INOCA. The preliminary logistic regression model, with internal validation and receiver operating characteristic (ROC) curve analysis, demonstrated moderate discrimination (area under the curve (AUC) = 0.70; 95% confidence interval (CI): 0.66–0.74; sensitivity = 79.80%; specificity = 54.12%). Calibration curve and decision curve analysis (DCA) suggested good predictive accuracy and clinical net benefit. (2) Using machine learning, a random forest model was constructed based on four features (SDNN, LDL-C, age, MONO#). Internal validation with ROC analysis showed excellent discrimination (AUC = 0.92; 95% CI: 0.89–0.95; sensitivity = 84.0%; specificity = 82.3%). (3) During the external validation phase, the logistic regression model showed limited performance (AUC = 0.59; 95% CI: 0.50–0.68; sensitivity = 58.92%; specificity = 58.24%), and the calibration curve and DCA curve showed only average model performance. In contrast, external validation of the random forest model yielded better discrimination (AUC = 0.71; 95% CI: 0.63–0.79; sensitivity = 84.5%; specificity = 53.0%).

**Conclusion:**

(1) Decreases in resting and stress left ventricular ejection fraction (LVEF) in patients with INOCA are more readily detected by PET/CT examination. (2) The risk of INOCA is closely associated with smoking history, SDNN, LDL-C, MONO#, and HCT. (3) Compared with multiple logistic regression analysis, the random forest prediction model shows more accuracy in predicting INOCA.

## 1. Introduction

Ischemia with no obstructive coronary arteries (INOCA)-related coronary microvascular disease (CMVD), also known as primary microvascular angina [1], refers to a clinical syndrome in which there are symptoms and objective evidence of myocardial ischemia, but no obstructive coronary artery disease (epicardial vascular stenosis ≥50%) is found on coronary CT angiography or invasive coronary angiography, and there is clear evidence of impaired coronary microcirculation function syndrome; the ultimate result is usually an increase in index of microcirculatory resistance (IMR) and a decrease in effective myocardial perfusion; occasionally, when the etiology is microcirculatory spasm, the corresponding parameters may also appear completely normal [2].

According to data from China, approximately 20% of patients hospitalized for angina and undergoing coronary angiography are diagnosed with INOCA [3]. Currently, the number of patients diagnosed with coronary heart disease in China can reach 11.39 million [4]. The burden of INOCA disease in our country is very high. Previously, INOCA was believed to have a good prognosis, but recent studies have shown that compared with asymptomatic patients, those with INOCA often have a poor prognosis, and the disease has a greater impact on their quality of life [5]. These findings indicate that our current understanding of this disease is insufficient, and larger-scale research is needed to help improve our diagnosis, understanding, and management of this challenging disease associated with adverse outcomes [6].

The main problem currently faced by INOCA is the difficulty of clear diagnosis, as it is not possible to visually detect the morphology and function of coronary microcirculation. It is generally believed that a decrease in coronary flow reserve (CFR) is a hallmark of CMVD in the absence of epicardial artery obstructive stenosis (lumen stenosis area greater than 50%). Positron emission tomography (PET) is a well-validated method and reference technique for the noninvasive quantitative measurement of myocardial blood flow (MBF) and CFR and may be considered as an option for the noninvasive assessment of myocardial flow, such as reserve transthoracic Doppler assessment of left anterior descending branch (LAD) flow reserve during stress echocardiography and CMR [7,8].

Although PET-based myocardial blood flow quantification is strongly correlated with the CFR obtained from invasive testing, it requires large amounts of equipment from hospitals and is difficult to popularize [9]. Therefore, exploring simple and feasible diagnostic methods to identify high-risk patients and avoid unnecessary PET will greatly promote the identification and investigation of INOCA.

Our study clarifies the grouping based on PET/CT, attempts to identify clinical indicators related to INOCA, and efforts to establish a preliminary INOCA prediction model to help clinical workers better identify patients with high-risk INOCA and screen this group of patients more selectively and in a targeted manner.

## 2. Object and Method

### 2.1 Research Object

#### 2.1.1 Inclusion and Exclusion Criteria

The inclusion criteria were as follows: age between 18 and 80 years; presence of angina pectoris or suspected symptoms of angina pectoris; and successful collection of individual basic information parameters.

The exclusion criteria were acute myocardial infarction within 1 week; high-risk unstable angina pectoris; severe heart failure; acute pulmonary embolism; acute pericarditis and myocarditis; adenosine usage contraindications; uncontrolled severe hypertension; previous revascularization surgery; previous diagnoses of myocardial infarction, cardiomyopathy, valve disease, congenital heart disease, or heart failure; malignant tumors; acute systemic diseases; uncontrolled metabolic diseases; severe lung diseases; severe liver and kidney dysfunction; and pregnant and lactating women.

#### 2.1.2 Selected Population for Model Construction

A retrospective analysis was conducted on 1723 patients who presented to our hospital between September 2019 and September 2022 due to chest pain and other symptoms, were suspected of having “angina pectoris” and were hospitalized; patients who completed nuclear myocardial flow reserve (CFR) measurement (drug load+resting dynamic tomography, using 13N-NH_3_ • H_2_O PET/CT coronary artery quantitative measurement) were screened. Among them, patients who underwent coronary angiography and had no obstructive lesions (stenosis degree <50%) and patients who met the following inclusion criteria and did not meet the exclusion criteria were ultimately included from among 755 cases.

#### 2.1.3 Model Validation Selected Population

From September 2022 to March 2024, patients who were admitted to our hospital with suspected “angina pectoris” due to chest pain and other symptoms were selected consecutively. These patients underwent coronary angiography, which revealed no obstructive lesions, and further complete CFR measurements. A total of 140 patients who met the inclusion criteria were ultimately selected.

### 2.2 Data Collection

Baseline data for all patients, including admission time, length of hospital stay, and the onset of symptoms at admission, were collected. The patient’s sex, age, height, weight (and body mass index (BMI)), hypertension history, diabetes history, hyperlipidemia history, smoking history, 19 blood routine tests, biochemical function indicators, five coagulation parameters, 24-hour dynamic electrocardiogram parameters, cardiac ultrasound parameters, and CFR parameters were collected.

### 2.3 Research Methods

#### 2.3.1 PET/CT CFR Examination

Prior to conducting PET/CT imaging, the use of vasoactive agents, calcium channel antagonists, dipyridamole, adenosine medications, theophylline medications, tea, coffee, or coffee-containing beverages was discontinued for a minimum of 24 hours. One-day stress + resting myocardial perfusion imaging was carried out: the imaging agent was administered in a resting state, and resting dynamic gated myocardial perfusion tomographic imaging was concurrently performed. For load imaging, adenosine was infused into the indwelling needle venous channel at a rate of 0.14 mg/kg/min, and the imaging agent was injected at the 3rd minute. During the peak load, the patient’s vital signs and discomfort were assessed, and dynamic gated myocardial perfusion tomographic imaging was initiated. The tomographic images are presented on the short axis (1–4 rows), vertical long axis (5–6 rows), and horizontal long axis (7–8 rows). From top to bottom, odd-numbered rows represent load images, whereas even-numbered rows represent resting images.

A PET/CT scanner utilizing 13N-NH_3_ • H_2_O as a tracer, which was generated by the GE Qilin Medical Cyclotron installed in our hospital, was used. Myocardial blood flow analysis was conducted on a Xeleris workstation (GE, USA) with software such as quantitative perfusion SPECT (QPS), quantitative gated SPECT (QGS), and Q.PET software Ver 2024 (Cedars Sinai, Los Angeles, CA, USA). By means of the 13N-NH_3_ • H_2_O myocardial blood flow pharmacokinetic model integrated into the software, the left ventricular vascular zones and overall MBF under resting and load conditions were automatically acquired, and the corresponding coronary flow reserve (CFR) was obtained; specifically, CFR = load MBF / resting MBF.

#### 2.3.2 Coronary Microcirculation Quantification (CFR) Examination Results

The 2022 Chinese Expert Consensus on the Diagnosis and Management of Ischemic Nonobstructive Coronary Artery Disease states that a CFR value <2.0 and the exclusion of the possibility of decreased myocardial blood flow reserve due to epicardial coronary artery obstructive disease suggest the presence of INOCA [3]. This guideline cites an article from the 2020 European Heart Journal [6]. The significance of the CFR values in this court is defined as a CFR value <2.0 being abnormal, values of 2.0–2.5 being critical, and a CFR value ≥2.5 being normal. Based on the CFR value determination method of our hospital, in this study, the minimum value of the CFR measured in the blood supply area of three coronary arteries was used as the basis for assessment. A CFR value <2.0 was defined as the INOCA group, a CFR value ≥2.5 was defined as the non-INOCA group, and a 2.0 ≤ CFR value < 2.5 was temporarily not included in the analysis (the “subhealthy” population) to more accurately compare the differences between the INOCA population and the healthy population.

#### 2.3.3 Statistical Methods

Statistical analyses were performed using SPSS statistical software (Version 26, Armonk, NY, USA: IBM Corp), Stata statistical software (17th Edition, College Station, TX, USA: Stata Corp LLC), and R language software (Version 4.4.1, R Foundation for Statistical Computing, Vienna, Austria). The acquired data contained missing values, which were processed using the multiple imputation method. Continuous variables are presented as the mean ± standard deviation, and categorical variables are presented as frequencies and percentages. An independent-samples *t* test was employed to compare the mean differences in various continuous variables between the diagnosed group and the control group. Under the null hypothesis that the difference in means between the two groups was zero, the test statistic was calculated, and the *p* value was obtained. If the *p* value was less than 0.05, the difference between the two groups was considered statistically significant. A chi-square test was used to compare the differences in the distribution of the two groups for categorical variables.

**Fig. 1.**
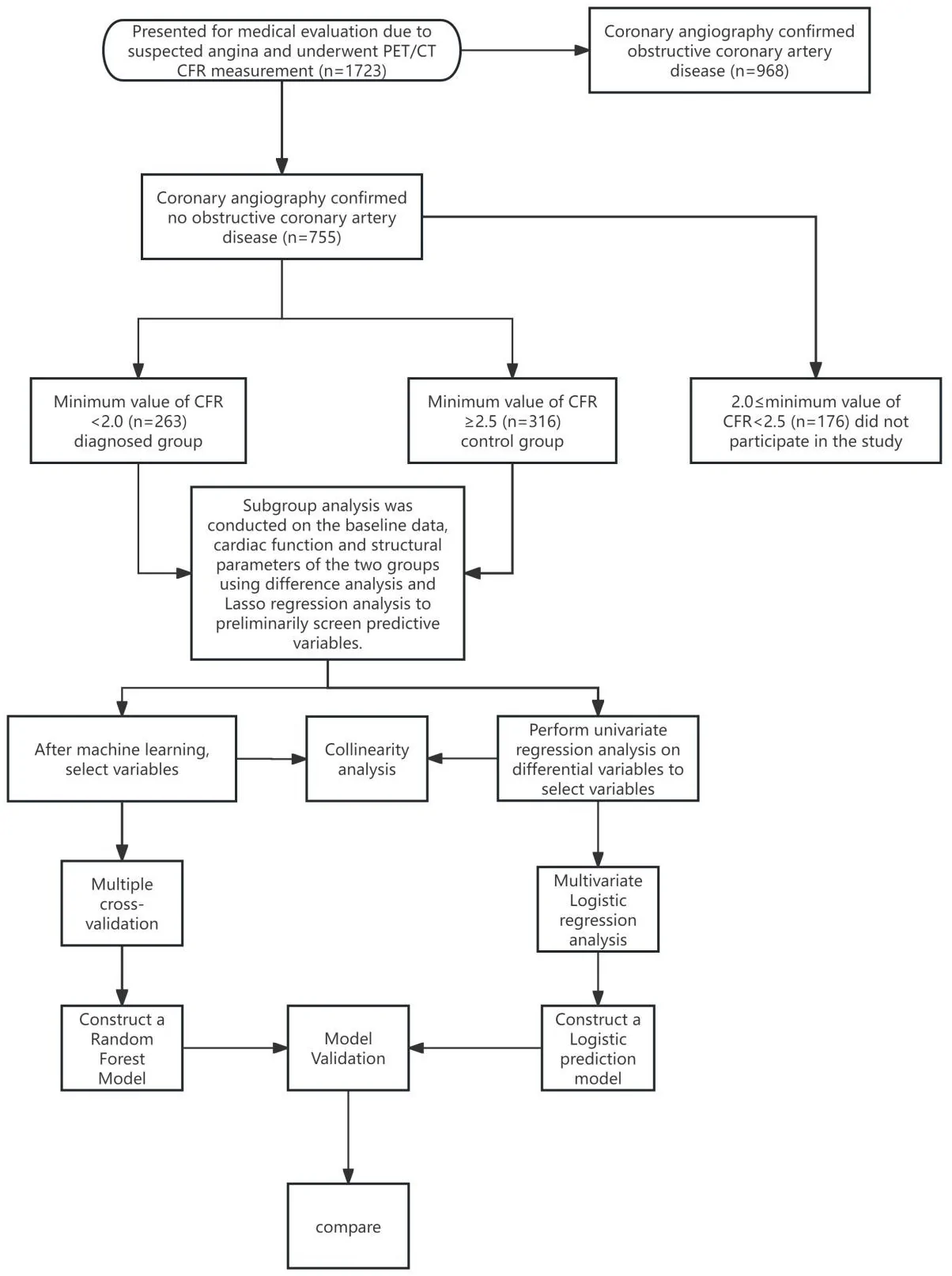
**Flowchart**. CFR, coronary flow reserve; PET, positron emission tomography.

First, a further univariate logistic regression analysis was carried out on the variables to assess the influence of each independent variable on the relationship between the diagnosed group and the control group. Multiple logistic regression analysis was subsequently performed to control for potential confounding factors. External data validation was conducted on the logistic regression diagnostic model, and the reliability of the model was evaluated by means of receiver operating characteristic (ROC) curves. Calibration and decision curve analysis (DCA) curves were plotted to evaluate the performance and clinical utility of the model. Moreover, a random forest model was employed to construct a diagnostic model and select important variables. Through adjusting the number of trees and feature selection, cross-validation (CV) was utilized to minimize the prediction error of the model. The curve depicting the change in the model error with respect to the number of trees and variables was plotted, and the variable with the highest predictive value was determined by ranking the feature importance. The cross-validation error curve and feature importance ranking of the model were used to assist in determining the optimal model parameters and key predictive factors, and ROC curve analysis was subsequently used to evaluate the performance of the constructed diagnostic model (Fig. 1).

## 3. Results

### 3.1 Overall Research-Related Analysis

During the model construction phase, a total of 755 hospitalized patients were included. The average age of the overall population, which included 471 females (62.38%) and 284 males (37.62%), was 60.45 years ± 9.61 years. According to the PET/CT coronary artery quantitative measurement results, according to the criteria of this study, there were a total of 263 patients (34.83%) in the diagnosis group and 316 patients (41.85%) in the control group.

During the model validation phase, a total of 140 patients were collected, including 108 females (77.14%) and 32 males (22.86%). Among them, 38 patients (27.14%) were diagnosed with INOCA, and 66 patients (47.14%) were in the control group.

All the continuous variables were tested for normality before further analysis. The normality of the data was verified through a combination of visual inspection and statistical testing. All the data conformed to a normal distribution or an approximately normal distribution.

#### 3.1.1 Demographic and Medical History Distribution

During the model construction phase, there was a significant difference in the distribution of smoking history between the diagnosis group and the control group, with smoking rates of 14.8% in the diagnosis group and 8.5% in the control group (χ^2^ = 5.61, *p* = 0.018).

During the model validation phase, the control group had a higher proportion of hypertension than did the diagnosed group (χ^2^ = 10.08, *p* = 0.018). See Table 1 for details.

**Table 1.** **Comparison of baseline data between the two groups (CFR of 2.0–2.5 excluded)**.

Variable		Model construction phase					Model validation phase				
		Total (n = 579)	Control group (n = 316)	Diagnosed group (n = 263)	t/χ^2^	*p*	Total (n = 104)	Control group (n = 66)	Diagnosed group (n = 38)	t/χ^2^	*p*
Age		60.45 ± 9.61	59.66 ± 9.72	61.41 ± 9.41	2.19	0.029	58.69 ± 9.58	58.74 ± 10.46	58.61 ± 7.97	0.07	0.944
BMI (kg/m^2^)		26.00 ± 3.94	25.98 ± 4.10	26.03 ± 3.75	0.14	0.888	26.73 ± 3.40	26.56 ± 3.76	27.01 ± 2.69	0.70	0.486
Hospitalization days		2.44 ± 1.46	2.39 ± 1.49	2.51 ± 1.42	0.99	0.324	2.69 ± 1.63	2.91 ± 1.85	2.32 ± 1.04	1.81	0.073
Sex					1.86	0.173				0.35	0.552
	Male	220 (38.00)	128 (39.63)	92 (34.98)			24 (23.08)	14 (21.21)	10 (26.32)		
	Female	359 (62.00)	188 (60.37)	171 (65.02)			80 (76.92)	52 (78.79)	28 (73.68)		
Angina pattern					0.71	0.871				1.02	0.796
	Rest angina	210 (36.27)	113 (35.76)	97 (36.88)			38 (36.54)	25 (37.88)	13 (34.21)		
	Exertional angina	185 (31.95)	104 (32.91)	81 (30.80)			33 (31.73)	21 (31.82)	12 (31.58)		
	Mixed angina	122 (21.07)	66 (20.89)	56 (21.29)			22 (21.15)	13 (19.70)	9 (23.68)		
	Anginal equivalents	62 (10.71)	33 (10.44)	29 (11.03)			11 (10.58)	7 (10.61)	4 (10.53)		
Hypertension					5.92	0.116				10.08	0.018
	No	256 (44.24)	148 (45.93)	108 (41.06)			48 (46.15)	27 (40.91)	21 (55.26)		
	Yes	323 (55.76)	168 (54.07)	155 (58.94)			56 (53.85)	39 (59.09)	17 (44.74)		
Diabetes					0.22	0.636				0.22	0.641
	No	455 (78.54)	246 (77.85)	209 (79.47)			74 (71.15)	48 (72.73)	26 (68.42)		
	Yes	124 (21.46)	70 (22.15)	54 (20.53)			30 (28.85)	18 (27.27)	12 (31.58)		
Hyperlipidemia					0.11	0.746				0.60	0.437
	No	379 (65.43)	205 (64.84)	174 (66.16)			55 (52.88)	33 (50.00)	22 (57.89)		
	Yes	200 (34.57)	111 (35.16)	89 (33.84)			49 (47.12)	33 (50.00)	16 (42.11)		
Smoking history					5.61	0.018				0.07	0.788
	No	513 (88.61)	289 (90.45)	224 (85.17)			98 (94.23)	63 (95.45)	35 (92.11)		
	Yes	66 (11.39)	27 (9.55)	39 (14.83)			6 (5.77)	3 (4.55)	3 (7.89)		

Note: BMI, body mass index; values are the mean ± sd for continuous variables and n (%) for categorical variables.

#### 3.1.2 Comparison of Cardiac Function and Structural Indicators

Population analysis during the model construction phase revealed that compared with the control group, the diagnosed group clearly had significantly lower CFR values. The load and resting left ventricular ejection fraction (LVEF), average heart rate (AHR), standard deviation of normal R-R interval (SDNN), the standard deviation of the average R-R interval of 5 minutes (SDANN), and SDNN/root mean square of successive difference in RR interval (RMSSD) ratio significantly differed between the two groups.

In the population analysis during the model validation phase, in addition to the CFR and resting LVEF, the left ventricular end diastolic diameter (LVEDD), AHR, SDNN and SDNN/RMSSD ratio also significantly differed. See Table 2 for details.

**Table 2.** **Comparison of cardiac function and structural indicators**.

Variable	Model construction phase					Model validation phase				
	Total (n = 579)	Control group (n = 316)	Diagnosed group (n = 263)	t	*p*	Total (n = 104)	Control group (n = 66)	Diagnosed group (n = 38)	t	*p*
LAD-CFR	2.71 ± 0.99	3.49 ± 0.52	1.78 ± 0.49	40.16	<0.001	2.91 ± 0.97	3.50 ± 0.66	1.88 ± 0.37	16.00	<0.001
LCX-CFR	2.53 ± 1.00	3.32 ± 0.55	1.59 ± 0.43	42.94	<0.001	2.61 ± 0.99	3.24 ± 0.64	1.53 ± 0.26	18.99	<0.001
RCA-CFR	2.87 ± 1.09	3.69 ± 0.65	1.88 ± 0.55	36.43	<0.001	3.09 ± 1.12	3.75 ± 0.79	1.95 ± 0.51	14.11	<0.001
Load LVEF (%)	63.99 ± 7.40	64.91 ± 6.69	62.89 ± 8.04	3.31	<0.001	62.82 ± 9.68	63.52 ± 10.99	61.61 ± 6.79	0.97	0.335
Resting LVEF (%)	64.97 ± 7.80	65.95 ± 7.04	63.78 ± 8.49	3.36	<0.001	62.80 ± 9.85	64.12 ± 10.95	60.50 ± 7.12	1.83	0.071
LVEF (%)	65.34 ± 5.41	65.68 ± 4.78	64.93 ± 6.06	1.67	0.096	64.87 ± 4.72	64.91 ± 4.96	64.79 ± 4.32	0.12	0.902
LVEDD (mm)	45.60 ± 4.17	45.37 ± 4.14	45.87 ± 4.18	1.45	0.147	45.24 ± 3.50	44.71 ± 3.83	46.16 ± 2.63	2.06	0.042
AHR (bpm)	69.50 ± 7.64	68.38 ± 7.42	70.74 ± 7.69	3.67	<0.001	69.12 ± 6.73	67.92 ± 7.30	71.18 ± 5.06	2.68	0.009
ASDNN (ms)	52.90 ± 18.16	52.98 ± 17.42	52.80 ± 19.05	0.12	0.904	54.29 ± 15.76	55.07 ± 18.56	52.93 ± 9.14	0.79	0.432
SDNN (ms)	117.13 ± 25.38	123.28 ± 24.61	109.73 ± 24.35	6.63	<0.001	117.52 ± 28.78	122.03 ± 29.65	109.67 ± 25.73	2.15	0.034
SDANN (ms)	101.96 ± 24.42	107.09 ± 24.54	95.80 ± 22.84	5.69	<0.001	102.63 ± 23.85	106.08 ± 23.46	96.64 ± 23.65	1.97	0.052
RMSSD (ms)	39.38 ± 20.87	40.12 ± 21.62	38.50 ± 19.94	0.93	0.353	38.43 ± 21.05	39.36 ± 24.68	36.81 ± 12.62	0.69	0.489
SDNN/RMSSD	3.47 ± 1.37	3.59 ± 1.38	3.32 ± 1.35	2.41	0.016	3.67 ± 1.53	3.93 ± 1.66	3.23 ± 1.16	2.53	0.013

Note: LAD, left anterior descending; LCX, left circumflex artery; RCA, right coronary artery; CFR, coronary flow reserve; LVEF, left ventricular ejection fraction; LVEDD, left ventricular end-diastolic diameter; AHR, average heart rate; ASDNN, SDNN index; SDNN, standard deviation of the R-R interval; SDANN, standard deviation of the average R-R interval of 5 minutes; RMSSD, root mean square of the R-R interval difference; values are the mean ± sd for continuous variables and n (%) for categorical variables.

#### 3.1.3 Comparison of Biochemical Coagulation Indicators

During the model construction phase, the average value of the low-density lipoprotein-cholesterol** (**LDL-C) diagnosis group was significantly greater than that of the control group. During the model validation phase, the average value of the lactate dehydrogenase (LDH) diagnosis group was significantly greater than that of the control group. See Table 3 for details.

**Table 3.** **Comparison of biochemical coagulation indicators**.

Variable	Model construction phase					Model validation phase				
	Total (n = 579)	Control group (n = 316)	Diagnosed group (n = 263)	t	*p*	Total (n = 104)	Control group (n = 66)	Diagnosed group (n = 38)	t	*p*
LDL-C (mmol/L)	2.77 ± 0.92	2.70 ± 0.88	2.87 ± 0.96	2.21	0.028	2.84 ± 0.99	2.83 ± 1.04	2.86 ± 0.91	0.11	0.912
LDH (U/L)	168.34 ± 32.35	167.97 ± 33.09	168.78 ± 31.49	0.30	0.763	167.50 ± 30.16	172.88 ± 32.84	158.16 ± 22.30	2.71	0.008

Note: LDL-C, low-density lipoprotein cholesterol; LDH, lactate dehydrogenase; values are presented as the mean ± sd for continuous variables and n (%) for categorical variables.

#### 3.1.4 Comparison of Blood Routine Parameters

Model construction stage study population: Among the numerous blood routine parameters, red blood cell-related parameters, including red blood cell count (RBC), mean corpuscular hemoglobin concentration (MCHC), hemoglobin (HGB) and hematocrit (HCT), significantly differed between the two groups. In addition, for monocyte absolute value (MONO#), the average value of the diagnosed group was significantly greater than that of the control group. There were no significant differences in the other indicators.

During the model validation phase, none of the blood routine parameters of the study significantly differed, as shown in Table 4.

**Table 4.** **Comparison of blood routine parameters**.

Variable	Model construction phase					Model validation phase				
	Total (n = 579)	Control Group (n = 316)	Diagnosed group (n = 263)	t	*p*	Total (n = 104)	Control group (n = 66)	Diagnosed group (n = 38)	t	*p*
RBC (×10^12^/L)	4.53 ± 0.46	4.57 ± 0.45	4.49 ± 0.46	2.27	0.023	4.50 ± 0.46	4.47 ± 0.51	4.55 ± 0.38	0.77	0.441
MCHC (g/L)	337.78 ± 11.08	338.63 ± 10.74	336.75 ± 11.40	2.04	0.042	329.13 ± 9.91	328.62 ± 9.06	330.03 ± 11.30	0.69	0.489
HGB (g/L)	138.22 ± 15.16	139.68 ± 14.88	136.46 ± 15.34	2.56	0.011	134.83 ± 14.36	134.32 ± 14.25	135.71 ± 14.70	0.47	0.636
HCT (%)	40.76 ± 4.59	41.22 ± 3.89	40.21 ± 5.26	2.63	0.009	40.95 ± 4.06	40.88 ± 4.27	41.07 ± 3.71	0.23	0.820
MONO# (×10^9^/L)	0.32 ± 0.13	0.31 ± 0.11	0.33 ± 0.14	2.01	0.045	0.33 ± 0.14	0.34 ± 0.15	0.32 ± 0.12	0.64	0.526

Note: RBC, red blood cell; MCHC, mean corpuscular hemoglobin concentration; HGB, hemoglobin, HCT, hematocrit; MONO#, absolute value of monocytes; values are the mean ± sd for continuous variables and n (%) for categorical variables.

### 3.2 Variable Selection and Model Establishment

#### 3.2.1 Logistic Regression Analysis

Univariate analysis revealed that the following variables were significantly correlated with disease status, age, history of smoking, AHR, SDNN, SDANN, the SDNN/RMSSD ratio, LDL-C and MONO#, RBC, MCHC, HGB, and HCT. See Table 5 for details.

**Table 5.** **Overall logistic regression analysis results**.

Variable	Univariable					Multivariable				
	b	SE	Z	*p*	OR (95% CI)	b	SE	Z	*p*	OR (95% CI)
Age	0.02	0.01	2.18	0.029	1.02 (1.01, 1.04)	0.01	0.01	1.38	0.241	1.01 (0.99, 1.03)
Smoking history	0.62	0.27	2.34	0.019	1.86 (1.11, 3.14)	0.58	0.29	2.00	0.036	1.81 (1.04, 3.16)
AHR (bpm)	0.04	0.01	3.59	<0.001	1.04 (1.02, 1.07)	0.01	0.01	0.60	0.481	1.01 (0.98, 1.04)
SDNN (ms)	–0.02	0.00	–6.15	<0.001	0.98 (0.97, 0.98)	–0.02	0.01	9.78	0.002	0.98 (0.96, 0.99)
SDANN (ms)	–0.02	0.00	–5.35	<0.001	0.98 (0.97, 0.99)	0.00	0.01	0.45	0.749	1.00 (0.99, 1.02)
SDNN/RMSSD	–0.15	0.06	–2.38	0.017	0.86 (0.76, 0.97)	–0.09	0.07	–1.25	0.221	0.91 (0.79, 1.05)
LDL-C (mmol/L)	0.20	0.09	2.19	0.028	1.22 (1.02, 1.46)	0.25	0.10	2.50	0.015	1.27 (1.05, 1.55)
RBC (×10^12^/L)	–0.42	0.19	–2.25	0.024	0.66 (0.45, 0.95)					
MCHC (g/L)	–0.02	0.01	–2.01	0.044	0.98 (0.97, 0.99)	–0.10	0.05	–2.11	0.430	0.99 (0.78, 1.01)
HGB (g/L)	–0.01	0.01	–2.53	0.012	0.99 (0.98, 0.99)					
HCT (%)	–0.06	0.02	–2.91	0.004	0.94 (0.90, 0.98)	–0.06	0.02	5.61	0.018	0.94 (0.90, 0.99)
MONO# (×10^9^/L)	1.57	0.78	2.01	0.044	3.76 (1.04, 20.4)	1.25	0.78	4.051	0.044	4.61 (1.04, 20.4)

Note: values are mean ± sd for continuous variables and n (%) for categorical variables.

Prior to conducting the multivariate analysis, a collinearity test was performed on the dynamic electrocardiogram parameters, and no collinearity was detected among the parameters. However, a collinearity test was conducted on the blood routine parameters, and significant collinearity was detected between HGB and HCT. Considering that there is also a certain correlation between RBC and HCT, and previous studies suggest that HCT may be related to CMVD, HGB and RBC in the blood routine parameters were not included in the multivariate regression analysis. Multiple logistic regression analysis was conducted on the remaining differential variables. After the influence of other variables was considered, only smoking history, SDNN, LDL-C, MONO#, and HCT remained significant after adjusting for other factors. See Table 5 for details.

Based on multiple logistic regression analysis, five independent related variables were selected, namely, smoking history, SDNN, LDL-C, HCT, and MONO#, which remained significant after adjusting for other factors. A logistic regression model was constructed using these five variables (Table 6).

**Table 6.** **Logistic regression model**.

Variable	b	SE	Z	*p*	OR (95% CI)
Constant term	4.04	1.09	13.8	0.000	
Smoking history	0.686	0.29	2.24	0.012	1.99 (1.14, 3.45)
SDNN (ms)	–0.02	0.00	35.4	<0.001	0.98 (0.97, 0.99)
LDL-C (mmol/L)	0.24	0.10	6.24	0.012	1.28 (1.05, 1.54)
MONO# (×10^9^/L)	1.66	0.04	5.65	0.017	5.26 (1.34, 20.7)
HCT (%)	–0.07	0.02	8.82	0.003	0.93 (0.89, 0.98)

Note: values are the mean ± sd for continuous variables and n (%) for categorical variables.

**Fig. 2.**
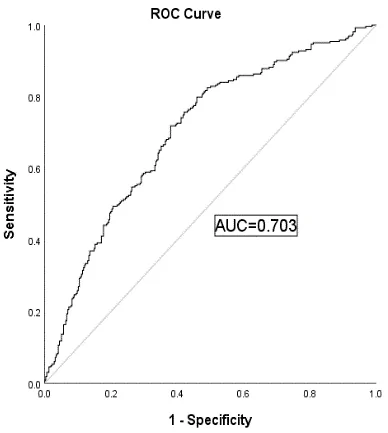
**Internal validation ROC curve**. ROC, receiver operating characteristic.

**Fig. 3.**
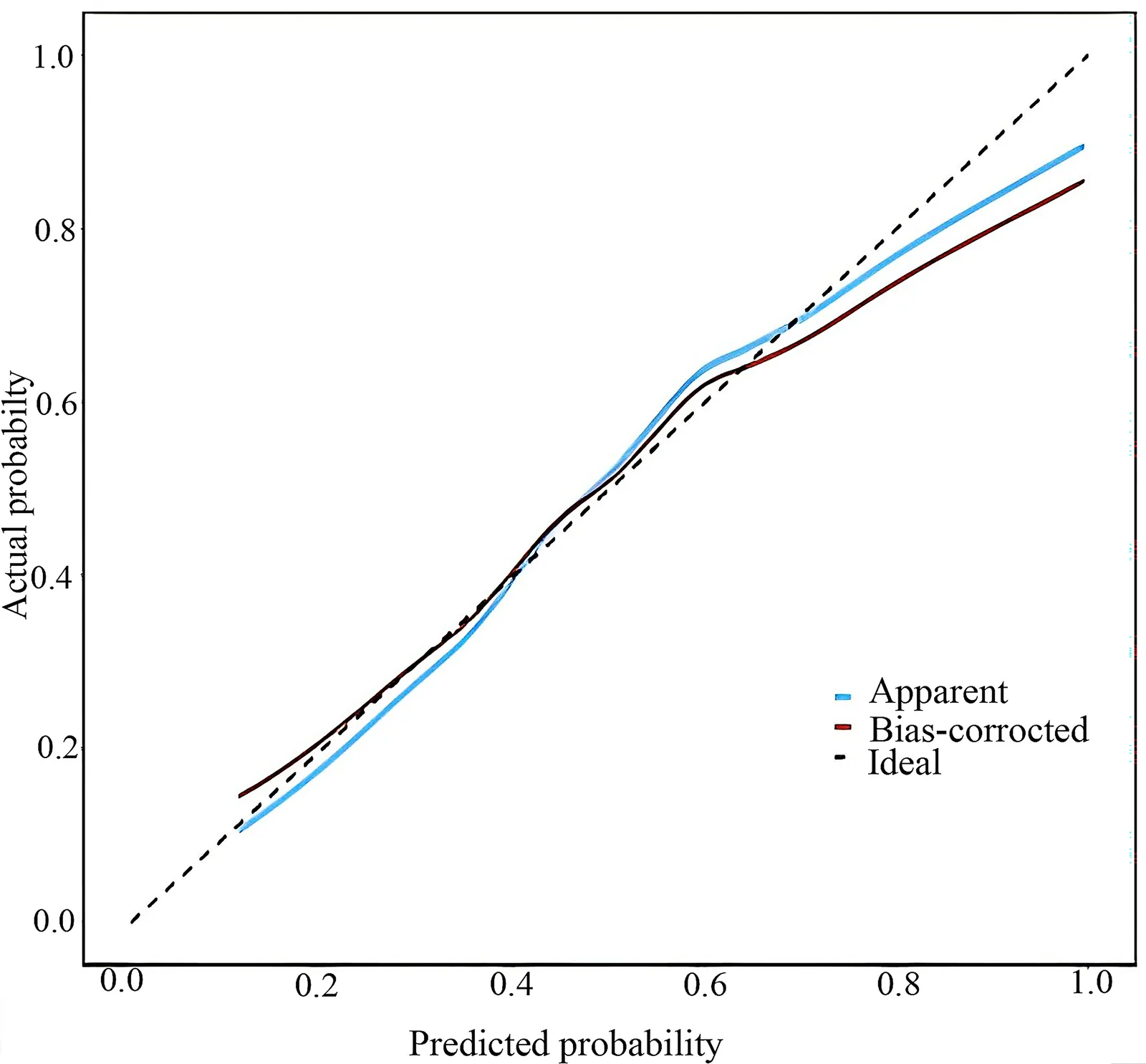
**Internal verification calibration curve**.

**Fig. 4.**
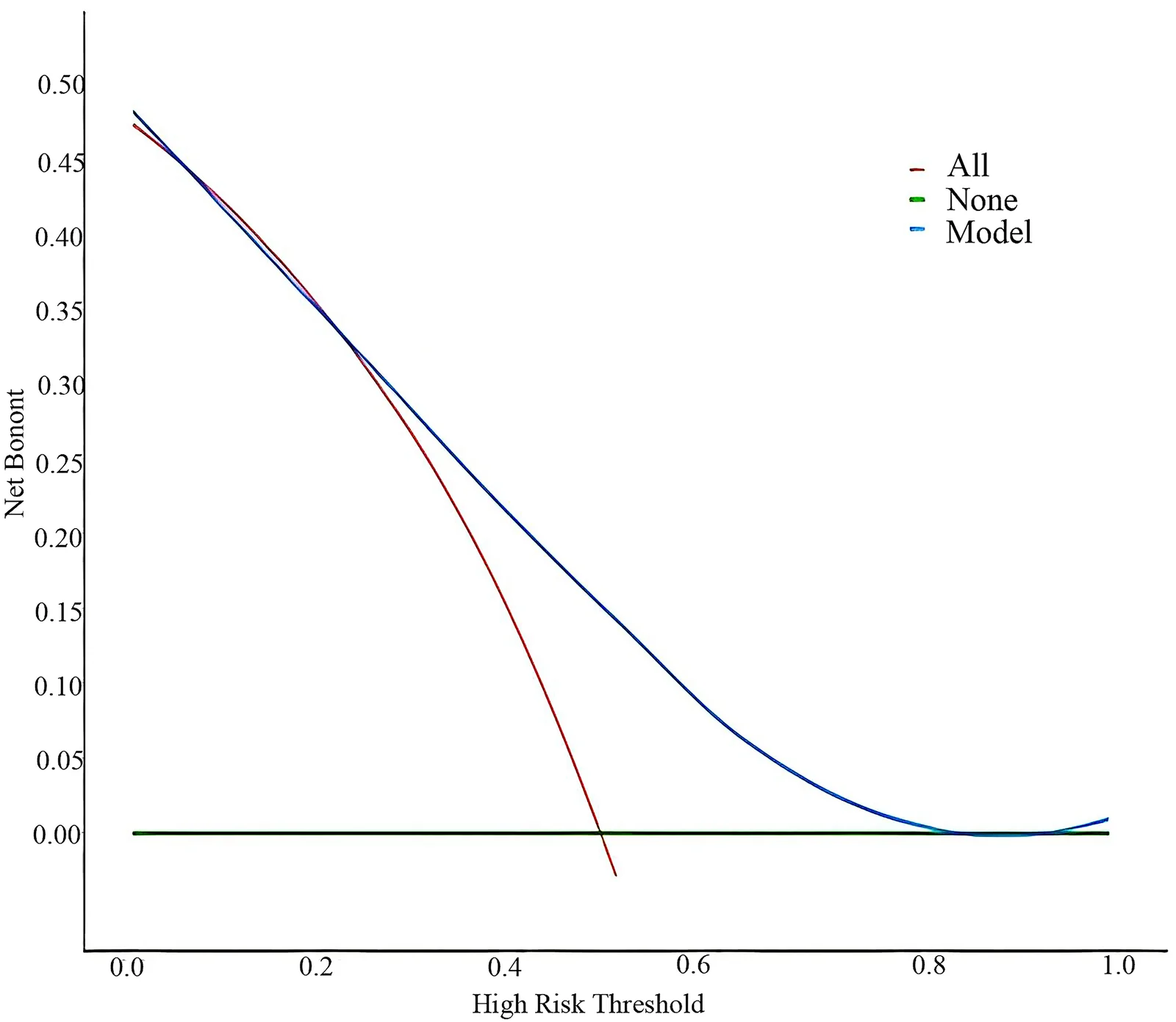
**Internal validation DCA curve**.

To preliminarily verify the accuracy and reliability of the model, as well as to determine the net clinical benefits of the model in practical decision-making, we conducted preliminary internal validation of the regression model and plotted the ROC curve and calibration curve and performed decision curve analysis (DCA) of the logistic regression model. The results revealed that the model accuracy was good (AUC = 0.70 (95% CI: 0.66, 0.74), sensitivity = 79.80%, and specificity = 54.12%), as shown in Figs. 2,3,4.

According to the calibration curve, the prediction accuracy between the actual probability and the model prediction probability was good, and there was a clear clinical net benefit.

#### 3.2.2 Establishment of the Random Forest Model

**Fig. 5.**
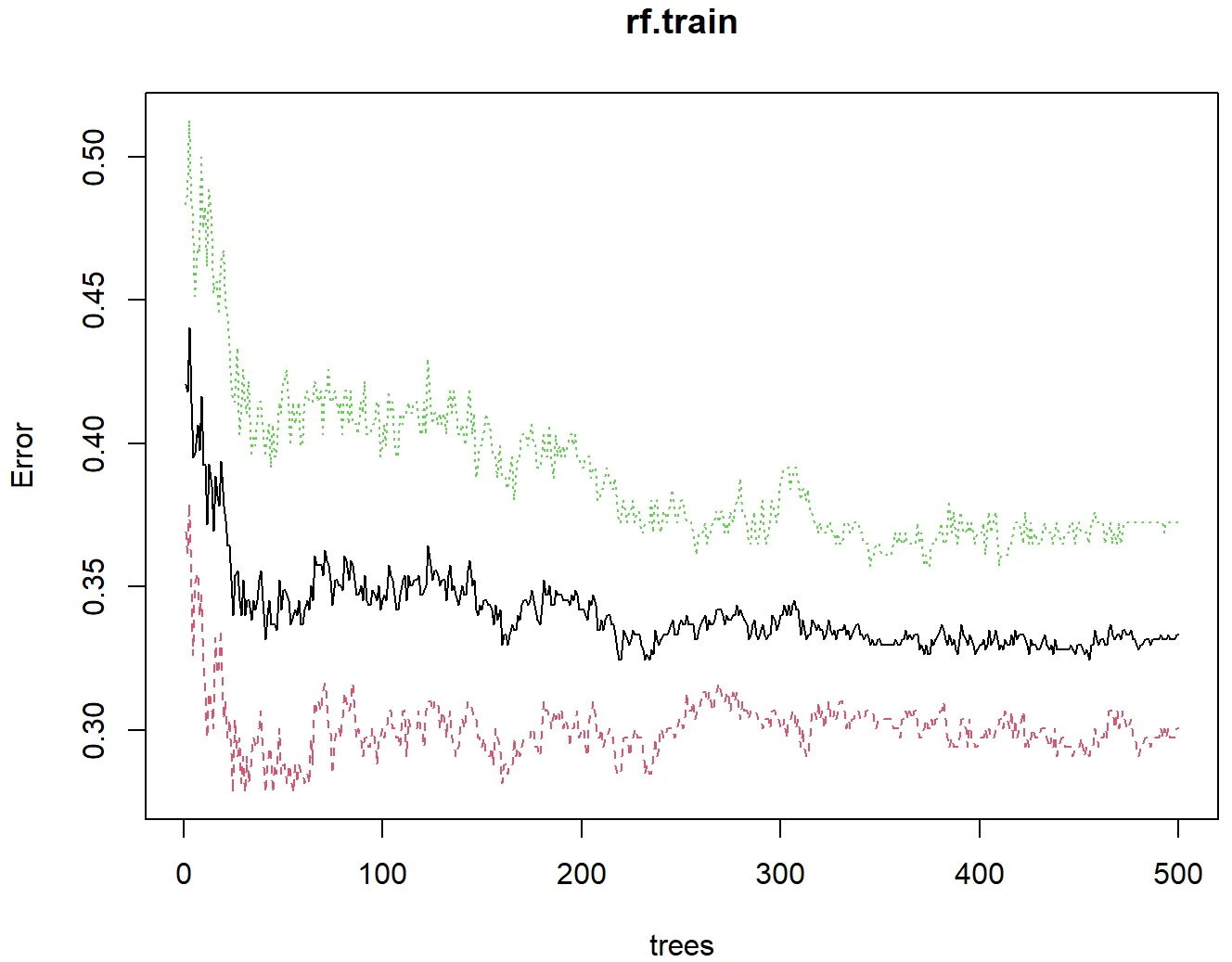
**Influence of random forest quantity on error**. Black: Typically represents the overall error rate of the random forest model. Red dashed line: Typically represents the error rate of the random forest model for one class. Green dotted line: Typically represents the error rate of the random forest model for another class.

A random forest model was then constructed using machine learning methods. First, the number of decision trees was determined. As shown in Fig. 5, as the number of trees increased, the error rate of the model gradually decreased until the number of trees reached a certain value, at which point the error rate tended to stabilize. According to the information in the figure, when the number of trees was 219, the error rate of the model was the lowest. Therefore, 219 trees were selected as the optimal parameters in the model.

**Fig. 6.**
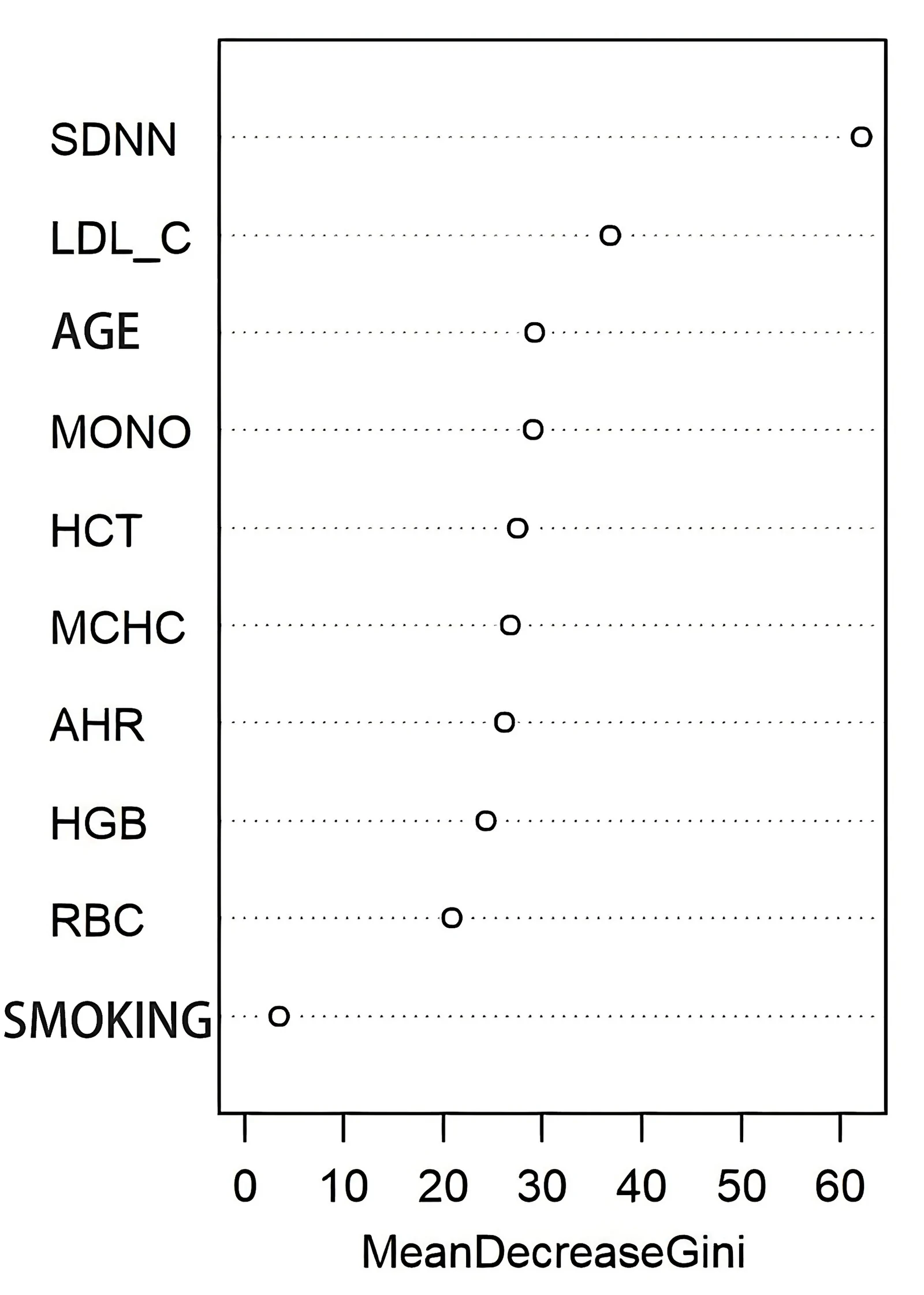
**Importance ranking of feature variables**.

After machine learning, the ranking of feature variables was obtained (Fig. 6) based on their mean decrease in the Gini index, which reflects the importance of each variable to classification decisions. The results show that the first four important feature variables were the SDNN, LDL-C, age and MONO#. These variables had the greatest impact on the prediction target in the model and therefore occupied important positions in analysis and prediction.

**Fig. 7.**
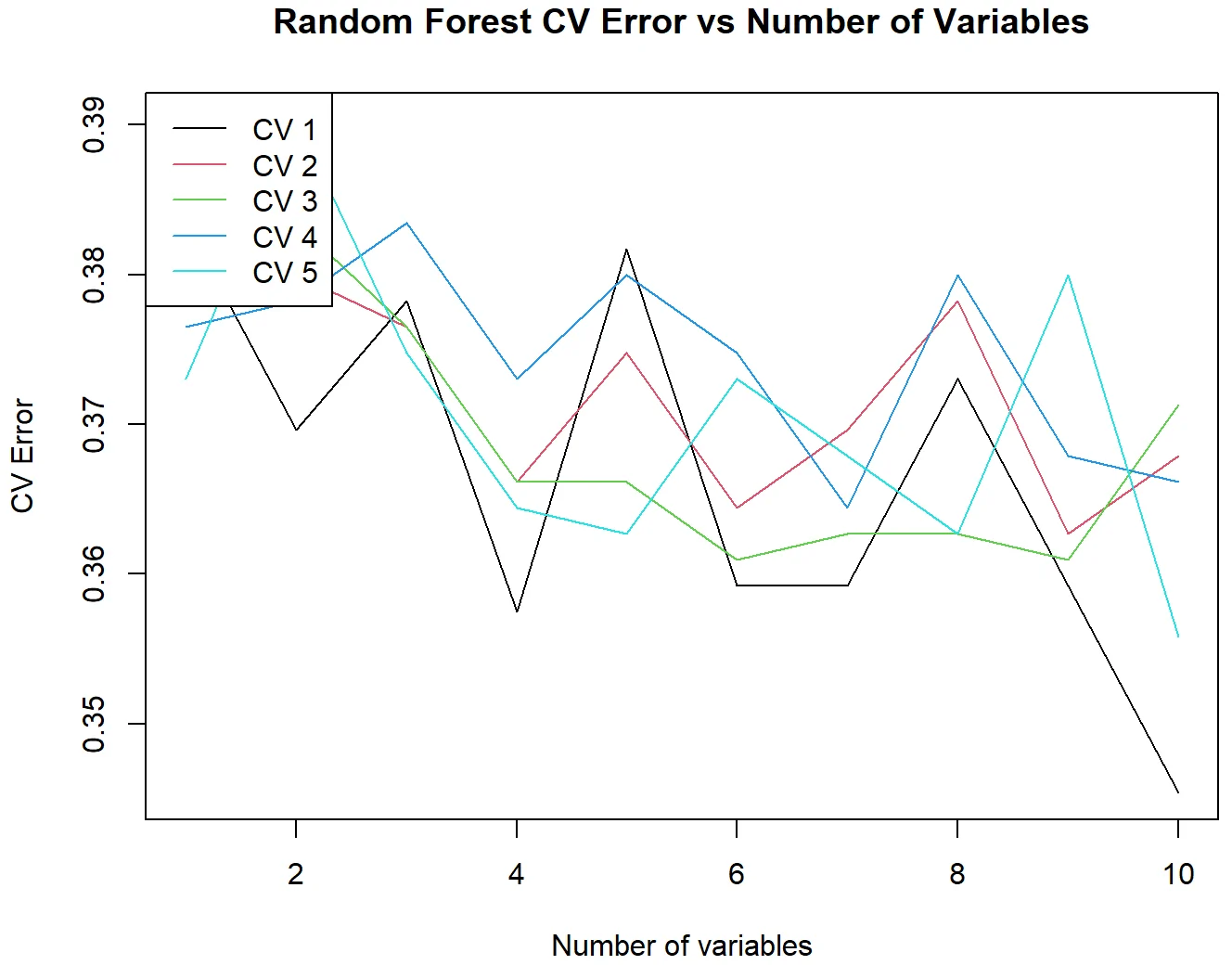
**Error rate curve and cross-validation plot of the random forest**. CV, cross-validation.

The variation in the cross-validation error rate of the random forest model under different feature selection quantities is shown in Fig. 7. As the number of variables increased, the cross-validation error rate tended to fluctuate. When the number of variables was 4, the error rate of the model reached its minimum; therefore, 4 features (SDNN, LDL-C, age, MONO#) were selected for modeling in the final model. This choice was validated through multiple replicate validations, ensuring the stability and performance of the model.

**Fig. 8.**
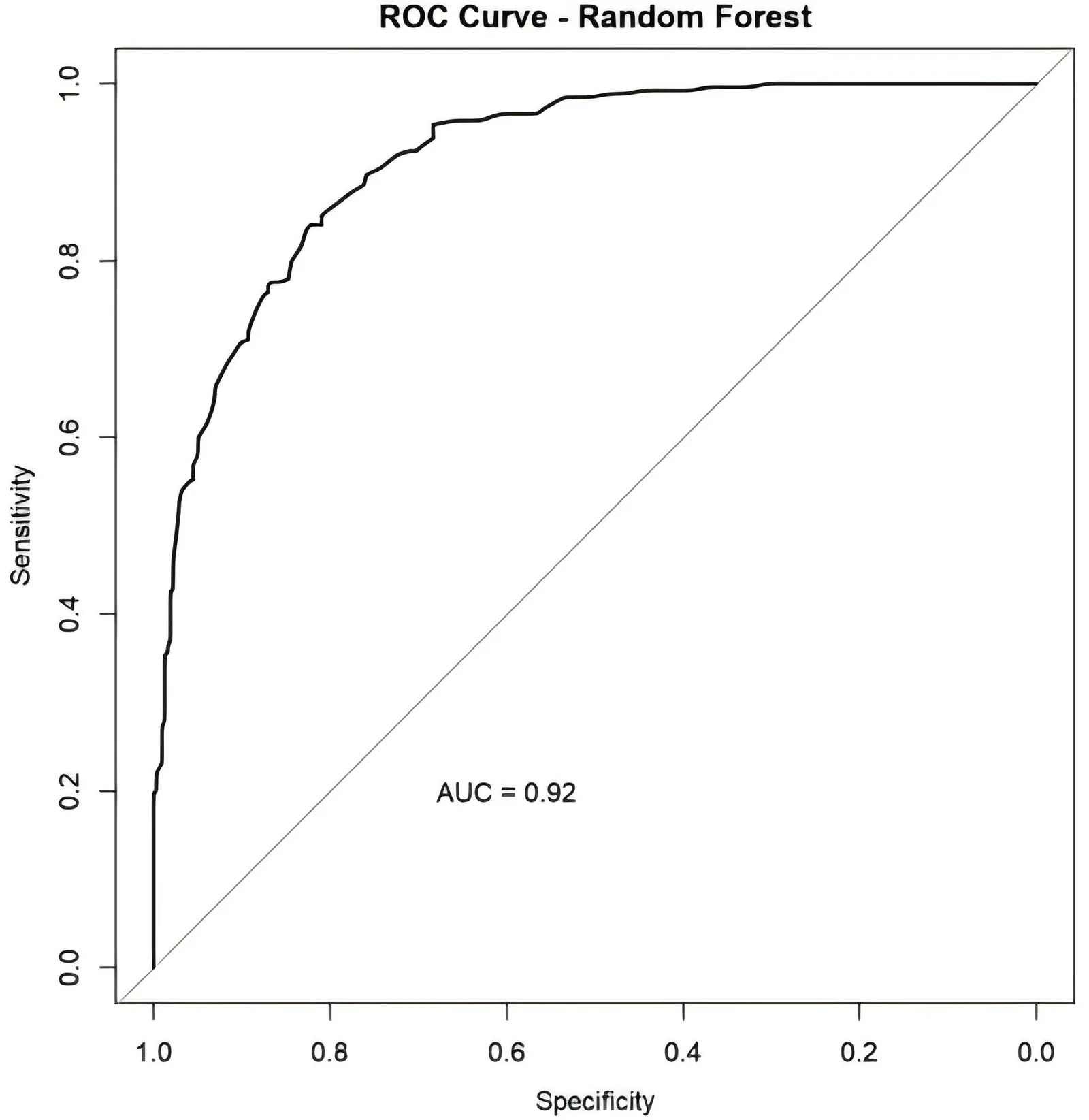
**ROC curve of the random forest**.

A random forest model was constructed based on the selected feature variables, and internal validation was conducted in the first part of the population. The ROC curve (Fig. 8) between the predicted values obtained and the actual state of the patient was plotted. The results revealed that the AUC was 0.92 (95% CI: 0.89, 0.95), the sensitivity was 84.0%, and the specificity was 82.3%.

### 3.3 External Validation of the Model

#### 3.3.1 External Validation of Logistic Regression Model

**Fig. 9.**
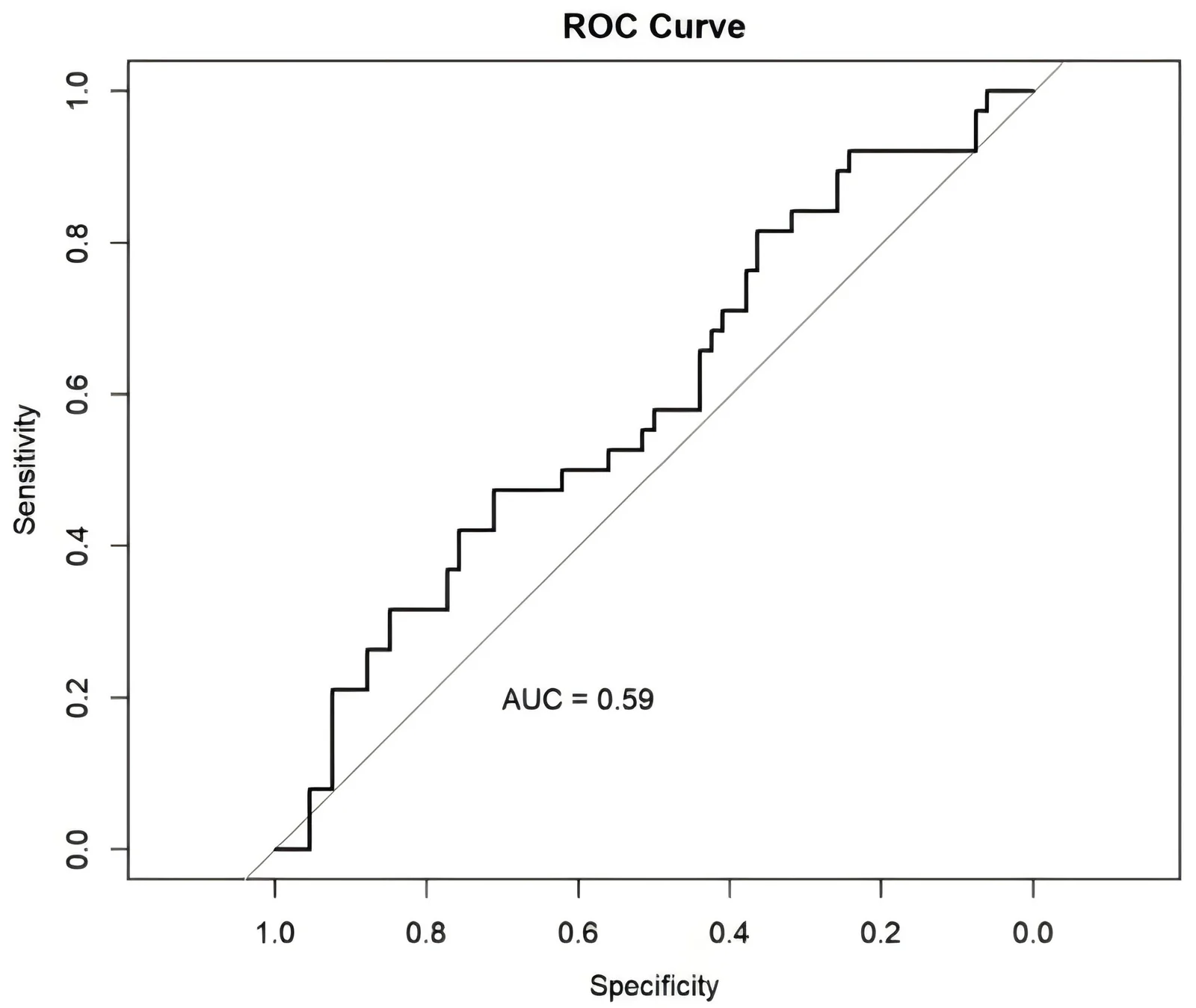
**ROC curve of the logistic regression model**.

In the overall multifactor analysis of the first part of the case, smoking history, SDNN, LDL-C, HCT, and MONO# remained significant after adjusting for other factors. A logistic regression model was constructed using these five variables, and external validation was conducted in the second part of the case. The predicted values obtained were used to plot the receiver operating characteristic (ROC) curve based on the actual state of the patient. The results revealed that the AUC was 0.59 (95% CI: 0.50, 0.68), the sensitivity was 58.92%, and the specificity was 58.24% (Fig. 9).

**Fig. 10.**
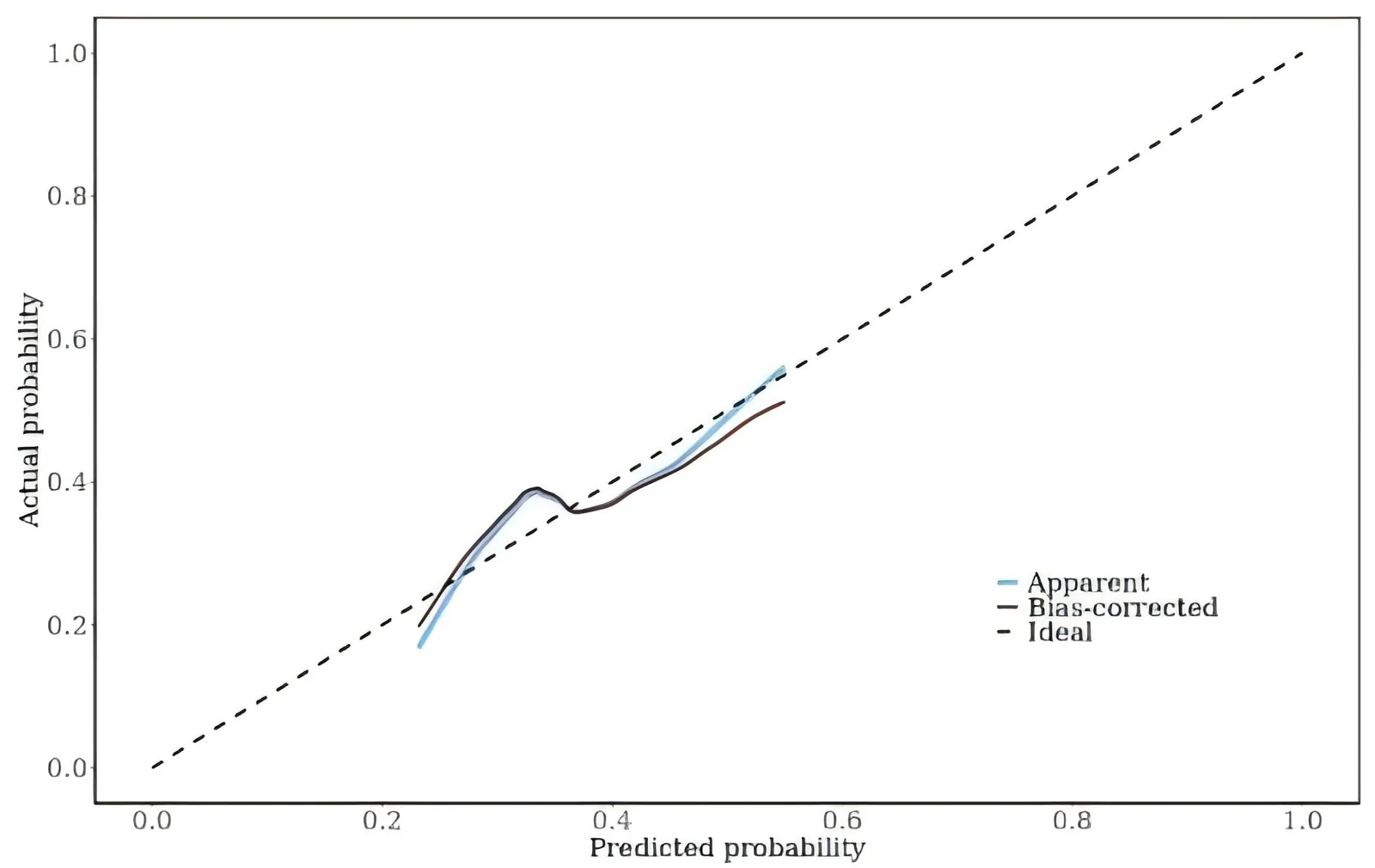
**External verification calibration curve**.

**Fig. 11.**
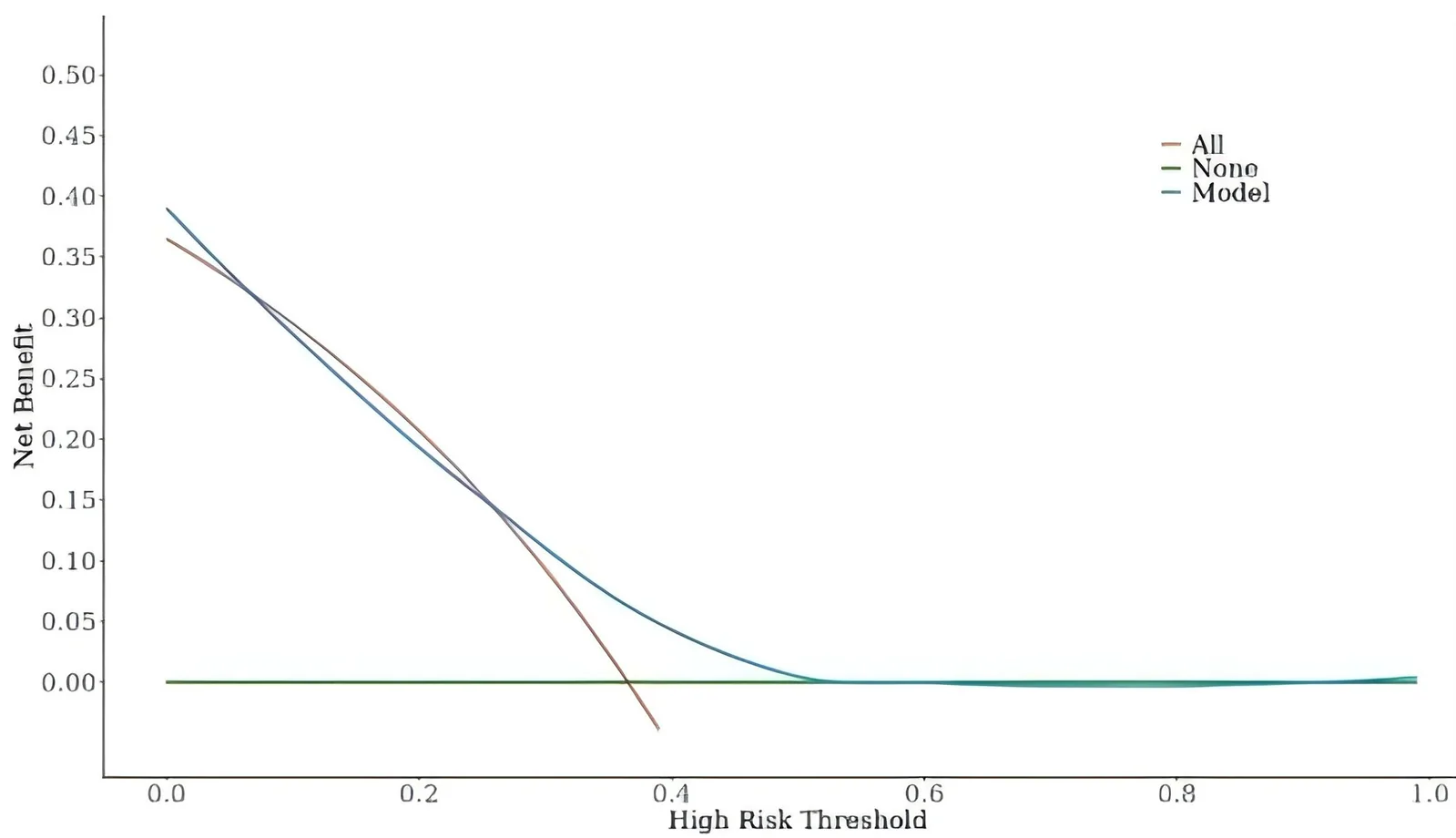
**External validation DCA curve**.

To further evaluate the predictive accuracy and reliability of the model and to determine the net clinical benefits of the model in practical decision-making, we plotted the calibration curve and decision curve analysis (DCA) for external validation of the logistic regression model, as shown in Figs. 10,11.

As illustrated by the calibration curve, the actual probability and the predicted probability of the model had average prediction accuracy. Compared with the internal validation model’s predictive ability and clinical net benefits, further improvement was needed.

#### 3.3.2 External Validation of the Random Forest Model

**Fig. 12.**
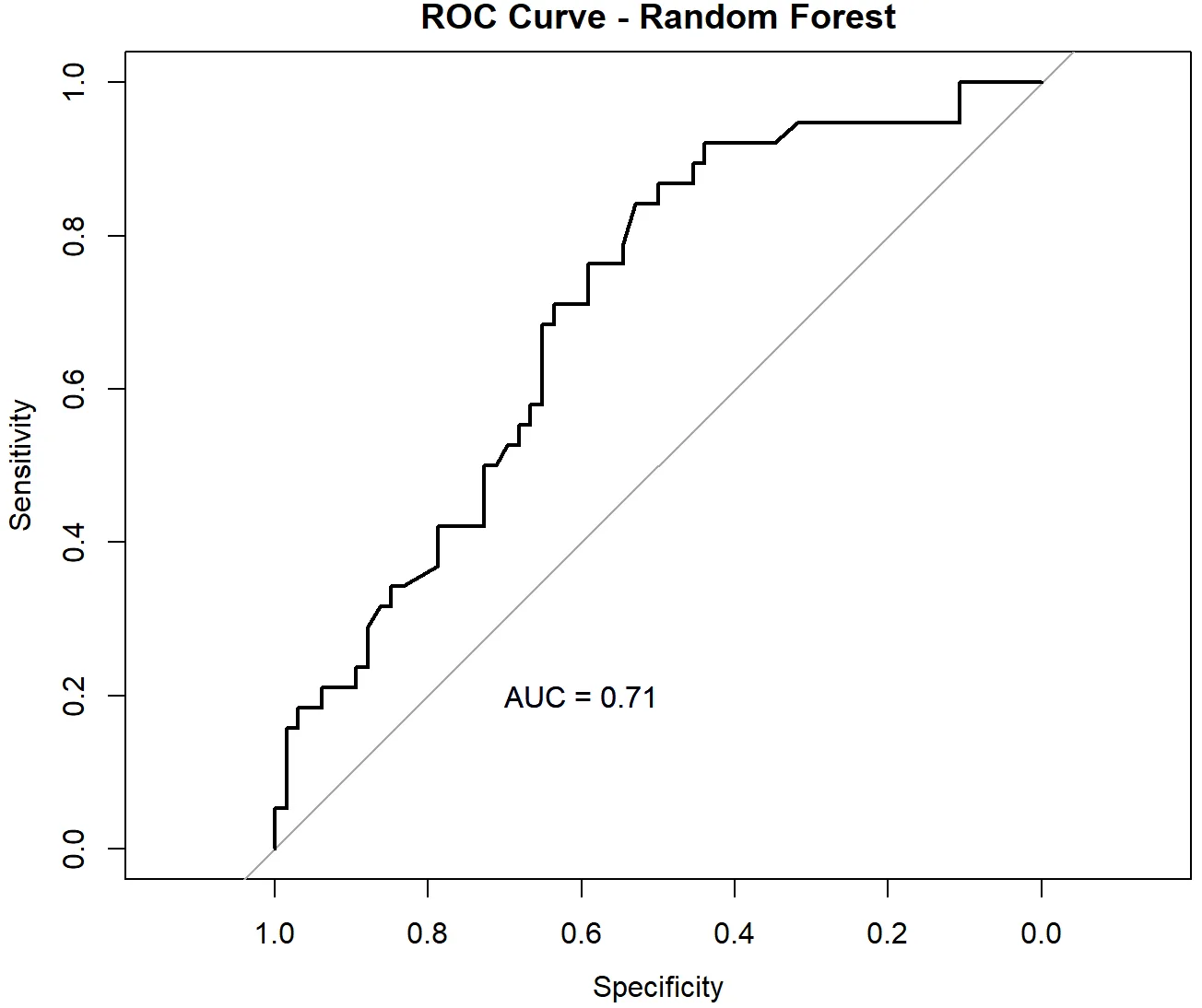
**External validation ROC curve of the random forest model**.

Using a random forest model for external validation, the ROC curve was plotted, and the results revealed that the AUC was 0.71 (95% CI: 0.63, 0.79), the sensitivity was 84.5%, the specificity was 53.0%, and the results were satisfactory, as shown in Fig. 12.

## 4. Discussion

Over the past 20 years, increasing evidence has shown that most patients with suspected INOCA (mostly females) have coronary vasomotor dysfunction or CMVD. Approximately half of patients with INOCA have CMVD, which was once thought to be a benign disease. However, later studies have shown that INOCA predicts adverse cardiac events and that patients with CMVD have a worse prognosis. Currently, CMVD is not fully diagnosed and treated due to the inconvenient nature of the diagnostic process, perhaps because clinical attention is focused more on patients with obstructive CAD [10].

In total, 755 patients suspected of having angina who underwent coronary angiography to confirm nonobstructive coronary artery disease and who subsequently underwent PET CFR examination were included in this study. In previous studies, the definitions of CFR varied within the range of 2.0–2.5, with some studies classifying it as abnormal and others as normal. Although recent domestic guidelines propose that a CFR <2.0 may indicate INOCA, certain studies adopt a CFR <2.5 as a criterion for CMVD, which might increase the rate of misdiagnosis while reducing the rate of missed diagnosis [11]. This study aimed to identify the factors associated with INOCA caused by CMVD. Therefore, a comparative analysis of patients with clearly diagnosed INOCA and those without confirmed myocardial ischemia should be conducted. A CFR within the range of 2.0–2.5 is defined as “subhealthy” and is not incorporated into the analysis of this study.

Previous studies and clinical work have confirmed that the proportion of patients with suspected INOCA is significantly greater among females [12,13,14]. We included 755 patients with suspected INOCA, 471 (62.38%) of whom were female. Among the 579 patients included in the study, 359 (62.00%) were female, which is similar to the previous assumption that women account for 2/3 of all patients. Among these 579 patients, 263 were diagnosed with INOCA (45.42%). Among the confirmed cases, 171 were female (65.02% of the total confirmed cases, 47.63% of the enrolled females), and 92 were male (34.98% of the total confirmed cases, 41.82% of the enrolled males). The data reveal a high proportion of women among patients with suspected INOCA. Although the percentage of women diagnosed with INOCA was slightly greater, the difference was not significant (47.63% vs. 41.82%, χ^2^ = 1.86, *p* = 0.173). This indirectly indicates that female patients often experience clinical discomfort related to cardiogenic factors, but the proportion of patients with confirmed CMVD is not significantly different from that of males, which has been proven in some domestic studies [15,16,17].

Traditional cardiovascular risk factors, such as sex, diabetes, hypertension, hyperlipidemia, smoking, obesity and aging [18,19,20,21,22,23], contribute to CMVD formation and development. Recent studies have shown that the inflammatory response and autonomic nervous system dysfunction may be important influencing factors [24,25,26]. In this study, patients with INOCA with CMVD and a control group without CMVD were analyzed. The results revealed that compared with the control group, the group of diagnosed patients had a greater proportion of smokers, were older, and had a 24-hour AHR that was 2.31 times/minute greater than that of the control group. In addition, their heart rate variability (HRV) parameter SDNN was lower, their LDL-C was higher, their red blood cell-related indicators in the routine blood test were lower, their monocyte number was higher, and their LVEF values measured by PET in the resting and load states were lower. These differences were statistically significant. However, there was no significant difference between the two groups in terms of history of three highs, i.e., liver and kidney function, electrolytes, and coagulation function. Multiple logistic regression analysis of differential indicators suggested that smoking, SDNN, LDL-C, MONO#, and HCT were independent factors associated with INOCA.

Previous study has reported the role of RBCs in coronary microcirculation [27]. As the main O_2_-transport carrier, RBCs may change function or undergo apoptosis under hypoxia-induced oxidative stress, increasing free radicals and altering their structure and function. Under hypoxia, RBCs have both functional damage and anti-hypoxia effects. During progressive hypoxia, RBC number and volume increase, glycolysis increases, antioxidant capacity increases, and NO is released to regulate blood flow for adaptation. Previous studies have shown that a moderate increase in HCT can improve cardiovascular function, as it increases vascular wall shear stress, stimulates NO release for vasodilation, improves microvascular function, and clears metabolic waste. However, insufficient vasodilation for HCT and increased blood viscosity may cause coronary slow flow (CSF). Excessive RBC apoptosis can exacerbate microcirculatory damage, bind to endothelial cells, stimulate clotting, and cause thrombosis. This study confirms that HCT is reduced in patients with INOCA and is an independent predictor of CMVD in patients with suspected INOCA.

This study confirmed a significant positive correlation between monocyte count (MONO#) and INOCA. Monocytes play important roles in *in vivo* inflammation and immune defense, secreting nonspecific esterases that recognize and kill bacteria, tumors and other cells. Other studies have shown that monocytes in acute Kawasaki disease patients may secrete cytokines, induce excessive endothelial cell autophagy, and participate in coronary artery endothelial cell [28] inflammatory damage, which is a possible mechanism underlying CMVD formation.

In the overall population, multiple variables were significant in the univariate analysis, but in the multivariate analysis, only smoking history, SDNN, LDL-C, HCT, and MONO# remained significant after adjusting for other factors, suggesting that they may be independent and closely related risk factors for CMVD. Moreover, subgroup analysis revealed that independent related factors vary across different specific populations, with a focus mostly on HRV-related parameters reflecting the autonomic nervous system (ANS), indicating that ANS dysfunction may play a more critical role in CMVD occurrence and development. Additionally, factors such as smoking status, mean platelet volume (MPV), HCT, uric acid (UA), and total bilirubin (TBIL), which reflect the inflammatory state, are significantly correlated, whereas conventional atherosclerosis risk factors contribute little.

## 5. Limitations and Prospects

First, this research is a single-center study, which inherently has selection bias and lacks true external validation. Second, the retrospective design inherently introduces the potential for selection bias and unmeasured confounding factors, as data were collected from existing clinical records rather than prospectively planned observations. This may affect the generalizability of the findings to broader populations. Third, the constructed predictive model shows acceptable stability but still offers room for improvement in performance. Fourth, the validation phase was constrained by objective factors (including the COVID-19 pandemic and diagnosis-related groups (DRGs)) and cost control measures, resulting in fewer enrolled patients than anticipated. Another issue that cannot be overlooked is the high cost of PET/CT, which restricts its availability at many centers and consequently limits the generalizability of research findings. Finally, given the complex etiology of INOCA, which results from multiple interacting factors, including not only coronary microcirculatory dysfunction but also pathogenic factors such as coronary microcirculatory spasm, PET scans do not consistently demonstrate reduced CFR in all patients with INOCA. Using CFR as the diagnostic criterion may consequently lead to the misclassification of some false-negative cases into the control group, thereby introducing statistical bias.

In subsequent studies, we will further optimize and enhance the performance of the research model and attempt to assign values, thereby stratifying patients through a scoring method. Doctors can quickly assess the likelihood of patients having INOCA using this model. For high-risk patients, PET/CT scans can be performed for confirmation, whereas low-risk patients do not require PET/CT scans, which helps to reduce overall diagnostic costs and avoid unnecessary overtesting. For centers lacking PET/CT facilities, the application value of this model is greater. It enables empirical diagnosis based on the model and clinical symptoms, guiding subsequent trial treatments without reliance on expensive PET/CT scans and thus avoiding unnecessary patient transfers to centers with PET/CT capabilities.

## 6. Conclusion

(1) Patients with INOCA have unique personal, lifestyle habits, and blood routine, biochemical, and cardiac functional characteristics; moreover, a decrease in static and load LVEF is more easily detected by PET/CT.

(2) INOCA is closely related to smoking history, SDNN, LDL-C, MONO#, HCT, and other indicators. Clinical screening of these indicators can help clarify the diagnosis of patients with INOCA.

(3) With respect to the SDNN and LDL-C, the random forest prediction model constructed with age and MONO# variables was more accurate at predicting INOCA diagnosis than the multiple logistic regression analysis prediction model was and has certain clinical application value.

## Data Availability

The datasets used and analysed in the present study are available from the corresponding author upon reasonable request.

## Abbreviations

AHR, average heart rate; ANS, autonomic nervous system; BMI, body mass index; CFR, coronary flow reserve; CMVD, coronary microvascular disease; CSF, coronary slow flow; DRG, diagnosis related groups; HCT, hematocrit; HGB, hemoglobin; HRV, heart rate variability; IMR, index of microcirculatory resistance; INOCA, ischemia with no obstructive coronary arteries; LAD, left anterior descending branch; LCX, left circumflex artery; LDH, lactate dehydrogenase; LDL-C, low-density lipoprotein-cholesterol; LVEDD, left ventricular end diastolic diameter; LVEF, left ventricular ejection fraction; MCHC, mean corpuscular hemoglobin concentration; MONO#, monocyte absolute value; MPV, mean platelet volume; NOCAD, nonobstructive coronary artery disease; PET, positron emission tomography; RBC, red blood cell count; RCA, right coronary artery; RMSSD, root mean square of successive difference in RR interval; SDNN, standard deviation of normal R-R interval; SPECT, single-photon emission computed tomography; TBIL, total bilirubin; UA, uric acid.
